# MRSA Prevalence and Risk Factors among Health Personnel and Residents in Nursing Homes in Hamburg, Germany – A Cross-Sectional Study

**DOI:** 10.1371/journal.pone.0169425

**Published:** 2017-01-09

**Authors:** Claudia Peters, Madeleine Dulon, Olaf Kleinmüller, Albert Nienhaus, Anja Schablon

**Affiliations:** 1 Institute for Health Services Research in Dermatology and Nursing, CVcare, University Medical Center Hamburg-Eppendorf, Hamburg, Germany; 2 Department of Occupational Health Research, Institution for Statutory Accident Insurance and Prevention in Healthcare and Welfare, Hamburg, Germany; Universidade de Lisboa Faculdade de Medicina, PORTUGAL

## Abstract

**Introduction:**

The increase of multidrug-resistant organisms in hospitals causes problems in nursing homes. Staff in geriatric nursing homes are at greater risk of MRSA colonisation. The aim of the study was to describe the occupational exposure to MRSA among health personnel in geriatric nursing.

**Methods:**

A point prevalence survey was conducted among health personnel and residents of geriatric nursing homes within the greater Hamburg district. Nasal swabs and, where relevant, wound swabs were collected for the screening survey. Risk factors for MRSA colonisation were identified by means of a questionnaire and using the files held on the residents. Where tests on nursing staff were positive, a control swab was taken; when the results were confirmed positive, decolonisation was performed. The responsible general practitioners were notified of positive MRSA findings among residents. A molecular biological examination of the MRSA samples was performed.

**Results:**

A total of 19 institutions participated in the study. Nasal swabs were taken from 759 nursing staff and 422 residents. Prevalence of MRSA was 1.6% among staff and 5.5% among residents. MRSA colonisation among health personnel indicated a correlation with male gender (OR 4.5, 95% CI 1.4–14.1). Among the residents, chronic skin diseases (OR 3.2, 95% CI 1.0–10.3) and indwelling devices (OR 3.2, 95% CI 1.2–8.1) were identified as risk factors. No link between MRSA in residents and in health personnel could be established.

**Conclusion:**

The number of MRSA colonisations among nursing staff and residents of geriatric nursing homes in Hamburg was rather low at 1.6% and 5.5% respectively and equates to the results of other surveys in non-outbreak scenarios.

## Introduction

Due to increases in life expectancy, a large number of elderly people are cared for in facilities for geriatric nursing. In 2013, 2.6 million people were in need of care in Germany, of which 29% were in full-time care in residential facilities. The need for care services rises with increasing age, resulting in a share of 64% of people aged 90 or above in need of nursing care [1].

Nosocomial infections are a particular problem in geriatric nursing homes. Elderly people are at greater risk of infection, for example as a result of chronic diseases and multimorbidity, weakened immune responses, limited mobility and frequent admissions to hospital [2].

A rise in multidrug-resistant organisms has been observed in hospitals [3]. Infections with resistant pathogens present not only the medical profession with a constant stream of new challenges, but also extend treatment times, increase mortality rates and raise treatment costs [4–7]. The situation with Methicillin-resistant *Staphylococcus aureus* (MRSA) is the best to examine, because this pathogen has presented the greatest problem in recent years. A continuous decline in the number of nosocomial MRSA infections is now being observed in Europe [8]. Current data from Germany has confirmed this trend, even if the precise causes for this are unclear [9].

An MRSA prevalence of 0.7% was found for the general population in Germany [10]. The frequency of MRSA colonisation among patients in the various healthcare fields has been specified at between 1% and 24% for Europe [11]. Among health personnel, average prevalence of 4.6% [12] and 5% [13] were found. A review on MRSA in non-outbreak settings showed prevalence between 0.2 and 15% [14]. Surveys of healthcare staff at medical institutions in Germany produced an MRSA prevalence of 0.4 to 4.5% [15].

Analyses conducted by the Statutory Accident Insurance of the Health and Welfare Service (BGW) have shown that the risk of infection is relevant for personnel. Out of 263 suspected cases of MRSA related occupational disease that were reported to BGW between 2010 and 2014, 39 cases of actual MRSA infection were recognized. Cases with merely MRSA colonisation do not meet the requirement for recognition as an occupational disease [16]. The risk of an occupational infection appears to be somewhat low on the whole. However, the consequences of infection can be severe, possibly resulting in many years of work incapacity and professional consequences [17]. Analysis of the BGW database on health personnel whose MRSA infection was recognised as an occupational disease showed that working as a geriatric nurse was one of the risks [18].

Little is presently known about the risk of occupational exposure to MRSA among geriatric healthcare workers in Germany. Therefore, a study was performed in which the point prevalence of MRSA colonisation among health personnel in geriatric nursing homes was surveyed. Occupational exposure was examined by screening the residents. Risk factors for MRSA colonisation were identified by means of a questionnaire. Potential links between MRSA colonisation in staff and residents were tested using genotyping.

## Methods

### Study design, setting and population

The cross-sectional study was conducted in geriatric nursing facilities throughout the greater Hamburg region. In these facilities, elderly people are cared for by qualified nursing staff 24 hours a day. 193 geriatric nursing facilities cared for 16,005 residents in Hamburg in 2013, with a total of 12,650 employees [19]. The nursing homes were recruited in writing by email, by telephone, and by providing information in the Hamburg network on multi-resistant pathogens, as well as by distributing leaflets at events. Because of the different methods used in the sampling procedure, the total number of facilities contacted to participate in the survey cannot be determined. The invitation to participate was directed at all employees and residents in nursing and care facilities in the Hamburg metropolitan region. An age range of 18 to 65 years was set as an inclusion criterion for nursing staff. The screening, consisting of sample collection and data acquisition, was performed from May 2014 to May 2015, and was conducted for a maximum of three days at each facility at short intervals.

### Data collection

For the MRSA survey, swabs were taken from the nasal vestibule of healthcare staff and residents. A wound swab was also taken from residents with chronic wounds. The study nurse performed the swab examination on the residents, whereas the health staff took their swabs themselves under the study nurse’s supervision. However, every tenth staff swab was repeated by the study nurse for quality control, in order to compare the self-administered sampling with the nurse-administered swabs. Potential risk factors for MRSA colonisation were identified using a questionnaire. For the staff, occupational risk factors such as the nature and duration of the work, contact with MRSA residents in a nursing capacity and factors of influence such as use of antibiotics, their own hospital stays and contact with animals were explored alongside socio-demographic data. For residents, data on age, degree of dependency of care (care level), chronic disorders, antibiotic therapies and hospital stays, as well as indwelling devices were collected. The nursing staff completed the questionnaire on their own, while the study nurse did this on behalf of the residents, adding medical data using the medical files and care records. The employee screening was anonymised with a coding system and no person-related data was recorded or kept in the study centre.

The participating health personnel were notified of their test results via sealed envelopes marked only with their identification codes, which were handed out by the care facilities management. Staff tested MRSA-positive were given the opportunity to contact the study nurse to obtain a control swab. If this control swab was still positive, the participant was provided with a non-antibiotic decolonisation kit consisting of hair and body wash, oral and nasal disinfectants and hand sanitizer with antimicrobial agents to eliminate MRSA from the body surface. They were also given products like tooth brushes and combs to avoid recontamination. The active ingredient in these products is Octenidine dihydrochloride. A further control swab was offered to check the success of the decolonisation efforts. The average time span between the screening and control swab was 14 days to 3 weeks. MRSA-positive results in residents were forwarded to general practitioners for further treatment.

### Microbiological methods

Cotton wool swabs were used for the nasal swab examinations. The swab sample was taken by swabbing both anterior nares in a rotating motion for around five seconds using the same swab. The swab was then sealed in a transport container. Immediately upon arrival of the material at the laboratory, the swab was first streaked onto an MRSA-selective plate (bioMérieux) and then placed into a Brain-Heart-Infusion enrichment broth (Becton Dickinson). Both, plate and broth were incubated at 37°C in an ambient atmosphere. The plate was inspected after 24 hours and 48 hours of incubation. Colonies were further identified by MALDI-TOF (Bruker Daltonics, MALDI Biotyper) either directly from the MRSA-selective plate when present as a pure culture or after isolation on CNA-Agar (Becton Dickinson). The presence of PBP2A was confirmed by an immunochromatographic assay (Alere, PBP2a SA test). After 24 hours of incubation, the enrichment broth was subcultered on a MRSA-selective plate, which was then incubated for another 48 hours with inspection after 24 and 48 hours. For positive samples, *S*. *aureus* protein A (*spa*) typing was performed. PCR amplification of the *spa*-gene was performed with the primers 1113f 5´-TAA AGA CGA TCC TTC GGT GAG C-´3 and *spa* 1514r 5´-CAG CAG TAG TGC CGT TTG CTT-´3 [20] using the Hot Start Taq Master Mix (Qiagen). Sequencing of the PCR product was carried out with the BigDye Terminator v3.1 (ThermoFisher) reagent. The sequencing reaction was then purified on Sephadex G-50 DNA Grade (ThermoFisher) columns and subsequently analysed in the ABI 3130xl Genetic Analyser. Resulting sequence data were interpreted with the ridom tool (http://www.spaserver.ridom.de/).

### Ethical approval

The study was conducted in accordance with the requirements of data protection legislation. The Ethics Committee of the General Medical Council for the city of Hamburg gave its approval (PV4610). The staff screening was anonymised with a coding system. An identification code was issued to the participants but this code was not linked to any identifying data. The sole purpose of this code was to transmit the lab test result to the participant by means of sealed envelopes that were handed out via the care facilities management. No person-related data or identification list was recorded or kept in the study centre or in the participating facilities. In accordance with the requirements of the local data protection legislation a written consent is not necessary for anonymised data collection and was omitted to avoid an unnecessary collection of personalised data. The health personnel gave their implicit consent verbally and by participation. The residents or their legal representatives had to give their written consent.

### Statistical analysis

The univariate analyses were performed using Pearson’s chi square test, or, where there were less than five observations, using Fisher’s exact test. Persons in whom MRSA was found were compared here against persons in whom it was not. For the multivariate analysis, backward stepwise logistic regression was applied. Variables with p-value >0.1 were successively excluded. The analyses were performed using IBM SPSS Statistics 22.

## Results

A total of 19 geriatric nursing facilities with 759 healthcare staff and 422 residents participated in the study. The response rate for all staff was 60%. In the individual facilities, between 35 and 95% of the staff participated in the study. For the residents, the response rate was 21% and between 7 and 56% in individual facilities.

### Health personnel

Over 80% of the participants were women (Table 1). The healthcare staff were between 17 and 65 years of age, with a median age of 43. The 50 to 59 age group was most strongly represented at 25%. Almost half of the staff had worked in geriatric care for between a full year and ten years, while 19% had worked in this field for more than 15 years. 60% stated that they had a qualification in care practice, with training as geriatric nurses and geriatric care assistants being the most commonly named. The majority of the personnel were engaged in care activities at the time of the survey, while others worked as physiotherapists, occupational therapists, speech therapists, social educators, social workers and other support personnel, domestic services and cleaning personnel, and also as management and administrative staff. Most health personnel worked on a general care ward or in dementia care. As part of their professional duties, almost three quarters specified that they had close contact with residents requiring care. Close contact was defined as the provision of basic care, facilitating resident mobility, treatment of bedsores and changing of bandages. In terms of personal risk factors, half of the staff specified that they had contact with pets or agricultural livestock. 34% of those surveyed had undergone antibiotic therapy in the last 12 months. Other risk factors such as hospital stays and/or surgical procedures in the last year, as well as chronic respiratory diseases and chronic skin diseases, were specified by 11% in each case. Nursing staff were only rarely involved in caring for relatives in their own private domestic environment or engaged in a secondary occupation in outpatient nursing care alongside their regular job.

**Table 1:** Description of study population (staff) and MRSA-positive cases (MRSA_staff_) among health personnel.

		Staff	MRSA_staff_	
Variable		N_total_ = 759	N_MRSA_ = 12	p-value^b^
		n (%)	n (%)^a^	
Sex	female	607 (80.0)	6 (1.0)	
	male	140 (18.4)	6 (4.3)	
	unknown	12 (1.6)	0 (0.0)	0.02
Age in years	< 30	164 (21.6)	4 (2.4)	
	30–39	163 (21.5)	4 (2.5)	
	40–49	178 (23.5)	0 (0.0)	
	50–59	191 (25.2)	2 (1.0)	
	> 60	53 (7.0)	2 (3.8)	
	unknown	10 (1.3)	0 (0.0)	0.25
Time spent in geriatric care	< 1 year	79 (10.4)	1 (1.3)	
	1–5 years	157 (20.7)	3 (1.9)	
	6–10 years	147 (19.4)	3 (2.0)	
	11–15 years	105 (13.8)	2 (1.9)	
	> 15 years	143 (18.8)	2 (1.4)	
	unknown	128 (16.9)	1 (0.8)	0.96
Level of training	geriatric nurse	241 (31.8)	6 (2.5)	
	care assistant / auxiliary nurse	110 (14.5)	2 (1.8)	
	general nurse	58 (7.6)	1 (1.7)	
	trainee nurse	45 (5.9)	1 (2.2)	
	without nursing qualification	78 (10.3)	1 (1.3)	
	other / unknown	227 (29.9)	1 (0.4)	0.64
Current occupation	active care / nursing work	471 (62.1)	10 (2.1)	
	physio- / occupational therapist	48 (6.3)	0 (0.0)	
	social worker	7 (0.9)	0 (0.0)	
	other / unknown	233 (30.7)	2 (0.9)	0.47
Close contact with patients		553 (72.9)	11 (2.0)	0.2
Contact with animals		396 (52.2)	6 (1.0)	1.0
Use of antibiotics		261 (34.4)	4 (1.5)	1.0
Hospital admission / surgical treatment		85 (11.2)	0 (0.0)	-
Chronic respiratory disease		83 (10.9)	0 (0.0)	-
Chronic skin disease		81 (10.7)	2 (2.5)	0.37
Caring for a dependant relative		38 (5.0)	2 (5.3)	0.12
Outpatient care		33 (4.3)	0 (0.0)	-

^a^ row percent.

^b^ comparison of MRSA positive against negative tested staff.

### Residents

The vast majority of residents at the nursing homes were female and over 80 years of age (Table 2). The participants of the study were mainly assigned to levels of care 1 and 2, while 22% were assigned to level 3 (most severe level). 19% had had a hospital stay or surgical procedure in the last three months, and 19% had taken antibiotics. 13% of the residents had indwelling devices such as a urinary catheter or a feeding tube, while 13% also had a chronic disorder of the respiratory tract. Only rarely were diabetes mellitus, chronic skin diseases, decubitus or chronic wounds and required dialysis found among the risk factors.

**Table 2:** Description of study population (residents) and MRSA-positive cases (MRSA_residents_) among residents in nursing homes.

		Residents	MRSA_residents_	
Variable		N_total_ = 422	N_MRSA_ = 23	p-value^b^
		n (%)	n (%)^a^	
Sex	female	301 (71.3)	15 (5.0)	
	male	121 (28.7)	8 (6.6)	0.49
Age in years	< 70	38 (9.0)	5 (13.2)	
	71–80	83 (19.7)	4 (4.8)	
	81–90	182 (43.1)	8 (4.4)	
	> 90	119 (28.2)	6 (5.0)	0.18
Level of care	not classified	14 (3.3)	1 (7.1)	
	care level 1	163 (38.6)	7 (4.3)	
	care level 2	151 (35.8)	9 (6.0)	
	care level 3	94 (22.3)	6 (6.4)	0.87
Use of antibiotics		82 (19.4)	7 (8.5)	0.18
Hospital admission / surgical treatment		80 (19.0)	7 (8.8)	0.17
Devices (urine catheter, gastric tube)		56 (13.3)	7 (12.5)	0.02
Chronic respiratory disease		53 (12.6)	4 (7.5)	0.51
Diabetes mellitus		41 (9.7)	2 (4.9)	1.0
Chronic skin disease		29 (6.9)	4 (13.8)	0.06
Decubitus / chronic wounds		15 (3.6)	3 (20.0)	0.04
Dialysis dependency		2 (0.5)	0 (0.0)	-

^a^ row percent.

^b^ comparison of MRSA positive against negative tested residents.

### MRSA prevalence

Nasal swabs were taken from 759 staff and 422 residents for examination for MRSA. 12 positives were found among the personnel (Table 1), putting the MRSA prevalence at 1.6% (95% CI 0.9–2.8%). The prevalence varied between 0 and 10.3% in the facilities under survey. Of those affected, ten were engaged in care duties, while eleven specified that they have close contact with residents in need of care. Two health personnel had chronic skin conditions, four had been treated with antibiotics, two cared for relatives at home, and six health personnel had a pet or contact with agricultural livestock. MRSA colonisation was found in six women and six men among the workforce. Four decolonisation treatments were performed in total, of which two were not successful. In a comparison of staff testing positive for MRSA against those testing negative, gender was the only factor that presented a statistically significant difference in the univariate analysis (p-value 0.02; Table 1). The logistic regression analysis generated an OR of 4.5 (95% CI 1.4–14.1) for male gender. A differentiated analysis in which only staff performing care duties were considered (n = 471) only generated a statistically significant result for men (OR 4.2, 95% CI 1.2–14.9) in terms of correlation between risk factor and MRSA colonisation.

Among the residents, 23 participants were tested MRSA-positive (Table 2), with a prevalence of 5.5% (95% CI 3.6–8.1%). The prevalence of MRSA ranged between 0% and 11.9% in the nursing homes. Eight wound swabs were also taken, of which one was MRSA-positive; this was for a resident whose nasal swab also tested positive. Seven of the residents with MRSA had a past hospital stay, seven had undergone a recent course of antibiotics, and seven had a urinary catheter or feeding tube. Four residents had a chronic skin disease, while four others had a chronic disorder of the respiratory tract. It was only possible to identify statistical significance in the univariate analysis for positive MRSA findings where decubitus, chronic wounds and the presence of devices were involved (Table 2). With logistic regression, an OR of 3.2 (95% CI 1.2–8.1) was derived for devices and 3.2 (95% CI 1.0–10.3) for chronic skin diseases. For other personal risk factors, there was no statistically significant association with positive MRSA findings among residents.

**Fig 1:**
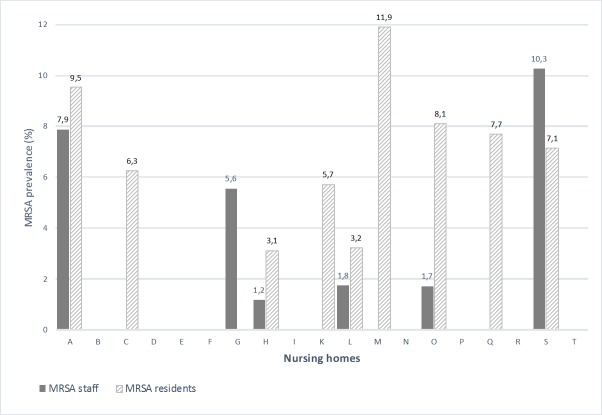
MRSA prevalence in 19 geriatric nursing homes in Hamburg. Prevalence is given as a percentage of total staff/residents screened and homes.

Distribution of MRSA findings varied greatly in the individual nursing homes (Fig 1). In five facilities, both personnel and residents were affected, in four nursing homes only residents were affected, and in one institution only staff were colonised. In nearly half of the participating nursing homes no MRSA was detected amongst staff or residents.

**Fig 2:**
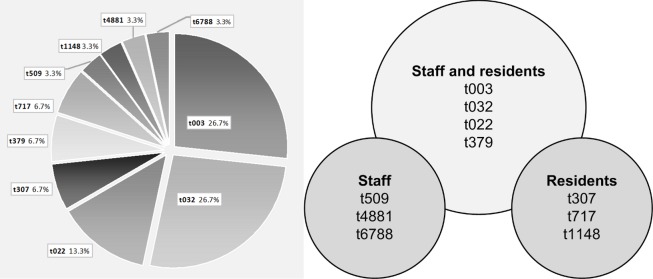
*Spa* typing: summary of the confirmed MRSA-positive results (A) and distribution of *spa* types among staff and residents (B).

The genotyping of the MRSA samples has shown mainly MRSA strains that occur commonly in Germany (Fig 2A). The Rhine-Hesse (t003) and Barnim (t032) epidemic strains were identified in more than half of the isolates. In the five facilities, in which staff and residents were affected, the MRSA strains differed in both groups. The distribution of *spa* types among the study population are given in Fig 2B.

## Discussion

The study on MRSA prevalence in geriatric nursing homes marks the first time that data has been available for health personnel and residents in the greater Hamburg district. It is also the largest survey to date of personnel working in geriatric care. MRSA colonisation rates of 1.6% were found in 759 personnel and 5.5% in 422 residents. The known risk factors for MRSA colonisation indicated a statistically significant association between MRSA colonisation and the male workforce. Among the residents, a correlation with chronic skin diseases and indwelling devices was identified.

Various studies are available on the MRSA colonisation of residents in geriatric nursing homes in Germany. More recent studies reported MRSA prevalence of between 2.3 and 9.2% [21–26]. In other European countries, lower prevalence of 0% and 0.3% was reported in Sweden [27] and the Netherlands [28] respectively, but there was also a higher MRSA prevalence of 12.2% in Belgium [29], 7.2% in Luxembourg [30] and 10.6% in Spain [31]. The Hamburg results for MRSA colonisation among residents of nursing homes in non-outbreak situations lie roughly in the middle of this range at 5.5%. A comparison of MRSA colonisation among health personnel with other studies is more difficult, because the studies mainly focused on hospital staff. For Germany, MRSA prevalence of between 0 and 7.7% has been specified for health personnel in geriatric nursing homes [32–35], while other countries report prevalence in the range of 5.8 to 14.5% [36–39]. However, the sample sizes for MRSA surveys of staff vary greatly. Many studies cover fewer than 100 participants.

Our analysis showed a more frequent MRSA colonisation among male staff, which was also indicated in other surveys [10, 40, 41]. Residents of geriatric care institutions frequently had indwelling devices, a risk factor for colonisation with MDROs [42] or MRSA [25, 34, 43] that was already evident in other studies.

For the MRSA samples, a molecular biological typing method was performed. This allows statements to be made on the frequency and distribution of certain MRSA clones and on the infection chain. First and foremost, the findings have shown the *spa* type t032 and t003 MRSA strains that commonly occur in Germany, as also reported by other studies [25, 32, 44, 45]. There was no evidence of possible transmission within a nursing home from resident to resident, resident to employee or vice versa. This was shown in part by the low number of positives found as well as by the variation of the strains within and among the institutions.

The staff that tested positive were first offered another control swab to eliminate the possibility of a merely short-term colonisation. If the test was again positive, the person affected was then provided with a standard Anti-MRSA Kit for decolonisation. They were then offered another swab to gauge the success of the decolonisation process. These options were only used by a few personnel, however. Only four persons in each case used the pre-/post-controls and the decolonisation treatment, and two health personnel remained MRSA-positive even after decolonisation. Both of them reported more than 10 years working in geriatric nursing, caring for a relative and contact with animals. The assigned occupational physicians handled further treatment.

In geriatric care facilities it is particularly important to find a balance between maintaining a sense of domestic well-being for residents and taking necessary medical or hygienic measures to prevent transmission of infections. Hand hygiene, for example, is an easy, cheap and effective way for self-protection and to prevent the transmission of pathogens [46]. In Germany, it is recommended to implement measures for a systematic compliance with standard infection control principles, which are normally adequate for preventing the spread of pathogens. Changes should be made on a case-by-case basis where known risk factors arise. For instance, isolation of MRSA-colonised or infected residents is not required as a general rule [47] and is not always an appropriate measure [48]. The described side effects of isolation have included severe depression and anxiety [49], especially in elderly people [50].

Routine screenings of personnel for MRSA is not recommended in Germany. However, in an outbreak situation affected health personnel can be examined. MRSA-positive staff are advised to perform decolonisation [15].

Different approaches were necessary to overcome initial difficulties in recruiting geriatric facilities. Face-to-face meetings were also held at institutions in the presence of relatives with a view to providing information and thus increasing the participation rate of residents. The reluctance of employers to agree is mainly attributed to the fear of numerous positive results. The frequently heard statement that MRSA-positive health staff would then take sick leave underlines the fear of the already real staff shortage in this sector. There were probably also concerns about reputational damage from high MRSA prevalence as well as increased workloads for personnel needed to prepare and support the screening. Ultimately though, the participation rate of healthcare staff in the participating institutions was a pleasing 60%.

The participation rate of residents was low, giving rise to the possibility that the results may have been distorted towards an underestimation of the actual MRSA risk. The participation procedure in accordance with data protection requirements clearly played a key role. Not all residents were able to sign the declaration of consent themselves. Guardianships as a result of dementia or other diseases may have impeded or prevented participation in the swab test.

Interest among the health personnel in the MRSA screening was much better, although they too may have been reluctant for fear of positive results. Adverse professional consequences such as stigmatisation, forced leave or mandatory professional retraining, especially where decolonisation is not possible, may have been just as critical as the fear of transmission in the domestic environment.

The lack of differentiation between transient and persistent MRSA carriage must also be mentioned as a limitation. Transient or intermittent carriers are people, who are colonised for short time periods, as opposed to persistent carriers, who are chronically colonised [12]. In this cross-sectional study only point prevalence was determined, hence a differentiation was not possible. Due to the anonymised screening method, MRSA positive staff members had to actively make contact with the study nurse to obtain a control swab. This option was only used by four personnel, as was the decolonisation results check using a new MRSA swab. The small number of positive results in our study means that it is not possible to make any valid statements on the success of the decolonisation measures.

## Conclusion

The results of the study of point prevalence in geriatric nursing homes in the greater Hamburg region indicated low MRSA prevalence among health personnel and residents. The rates are consistent with those from other studies in non-outbreak situations. The male gender was identified as a statistically significant risk factor for MRSA colonisation in this study. Among residents, the presence of chronic skin diseases and indwelling devices were shown as risk factors. The results provide a good basis for describing the MRSA risk of occupational exposure by health personnel in nursing homes.
